# Which measures of cigarette dependence are predictors of smoking cessation during pregnancy? Analysis of data from a randomized controlled trial

**DOI:** 10.1111/add.13395

**Published:** 2016-05-06

**Authors:** Muhammad Riaz, Sarah Lewis, Tim Coleman, Paul Aveyard, Robert West, Felix Naughton, Michael Ussher

**Affiliations:** ^1^ Population Health Research Institute, St George's University of LondonLondonUK; ^2^ Division of Epidemiology and Public Health and UK Centre for Tobacco and Alcohol StudiesUniversity of NottinghamNottinghamUK; ^3^ Division of Primary Care and UK Centre for Tobacco and Alcohol StudiesUniversity of NottinghamNottinghamUK; ^4^ Nuffield Department of Primary Care Health SciencesUniversity of OxfordOxfordUK; ^5^ Health Behaviour Research Centre, Department of Epidemiology and Public Health, UCLLondonUK; ^6^ Behavioural Science Group, Institute of Public HealthUniversity of CambridgeCambridgeUK

**Keywords:** Cigarette dependence measures, physical activity, predictors, pregnancy, randomised control trail, secondary analysis, smoking cessation

## Abstract

**Aims:**

To examine the ability of different common measures of cigarette dependence to predict smoking cessation during pregnancy.

**Design:**

Secondary analysis of data from a parallel‐group randomized controlled trial of physical activity for smoking cessation. The outcomes were biochemically validated smoking abstinence at 4 weeks post‐quit and end‐of‐pregnancy.

**Setting:**

Women identified as smokers in antenatal clinics in 13 hospital trusts predominantly in southern England, who were recruited to a smoking cessation trial.

**Participants:**

Of 789 pregnant smokers recruited, 784 were included in the analysis.

**Measurements:**

Using random‐effect logistic regression models, we analysed the effects of baseline measures of cigarette dependence, including numbers of cigarettes smoked daily, Fagerström Test of Cigarette Dependence (FTCD) score, the two FTCD subscales of Heaviness of Smoking Index (HSI) and non‐Heaviness of Smoking Index (non‐HSI), expired carbon monoxide (CO) level and urges to smoke (strength and frequency) on smoking cessation. Associations were adjusted for significant socio‐demographic/health behaviour predictors and trial variables, and area under the receiver operating characteristic (ROC) curve was used to determine the predictive ability of the model for each measure of dependence.

**Findings:**

All the dependence variables predicted abstinence at 4 weeks and end‐of‐pregnancy. At 4 weeks, the adjusted odds ratio (OR) (95% confidence interval) for a unit standard deviation increase in FTCD was 0.59 (0.47–0.74), expired CO = 0.54 (0.41–0.71), number of cigarettes smoked per day 0.65 (0.51–0.84) and frequency of urges to smoke 0.79 (0.63–0.98); at end‐of‐pregnancy they were: 0.60 (0.45–0.81), 0.55 (0.37–0.80), 0.70 (0.49–0.98) and 0.69 (0.51–0.94), respectively. HSI and non‐HSI exhibited similar results to the full FTCD.

**Conclusions:**

Four common measures of dependence, including number of cigarettes smoked per day, scores for Fagerström Test of Cigarette Dependence and frequency of urges and level of expired CO, all predicted smoking abstinence in the short term during pregnancy and at end‐of‐pregnancy with very similar predictive validity.

## Introduction

Smoking in pregnancy is the main preventable cause of poor birth outcomes, including miscarriage, stillbirth, prematurity and low birth weight 1, 2, 3, 4, 5, 6. Smoking also presents immediate risks for the mother, including placental abruption 7, as well as the long‐term risks reported for smokers in general. In high‐income countries, the prevalence of smoking during pregnancy is estimated to be between 10 and 26% 8, 9, 10, 11, 12, 13. It has been shown that smoking cessation during pregnancy improves maternal and fetal health and birth outcomes 14.

To target interventions for maternal smoking cessation appropriately, there is a need to identify which characteristics of smokers promote or inhibit smoking cessation during pregnancy 15, 16. A literature review 17 revealed a wide range of socio‐demographic, smoking and psychological characteristics investigated as potential predictors of smoking cessation during pregnancy. Socio‐demographic factors that have been shown to predict cessation significantly during pregnancy include maternal age, being married or living with partner, primiparity and higher socio‐economic status (income, education, housing, employment). Smoking‐related variables that have been found to predict cessation significantly in pregnancy include lower number of cigarettes smoked per day and if a partner or house member smokes. Finally, psychological variables that have been shown to predict cessation in pregnancy include lower levels of depression, stress and anxiety 17, 18. Other predictors of cessation include higher self‐efficacy for quitting, exposure to environmental tobacco smoke, exposure to patient education methods, greater perceived social support, stressful life events in early pregnancy, ethnicity, family history of diabetes and no use of marijuana before the pregnancy 19, 20, 21, 22.

Cigarette dependence measures have been shown to be valid in non‐pregnant smokers 23, 24, 25, 26, 27, but little is known about their validity for predicting smoking cessation in pregnancy. For example, among pregnant smokers the odds of cessation have been related inversely to baseline cotinine level 24, and in another study 29 scores for Fagerström Test of Cigarette Dependence (FTCD), urges to smoke and withdrawal symptoms failed to predict smoking status 2 weeks following the quit date. Therefore, in this study we examined the predictive validity of common measures of dependence on smoking cessation in pregnancy. As a demonstration of predictive validity, we expect that higher scores of these measures would be associated inversely with cessation. The most widely used measure of cigarette dependence is the FTCD 30, 31, 32, 33, while the biochemical marker of expired carbon monoxide (CO) 34, 35 and urge to smoke 36, 37 are also used commonly to measure dependence. The Heaviness of Smoking Index HSI 38, composed of two items from the FTCD (time to first cigarette of the day and number of cigarettes usually smoked per day), has been shown to predict failure of quit attempts in non‐pregnant smokers in both population‐based 24, 37 and clinical studies 27, 31, 32, 39. Therefore, we also examined the HSI and non‐HSI (comprised of the other four items in the FTCD) as predictors of abstinence. Urges to smoke have also been reported as significant predictors of abstinence in non‐pregnant smokers 37, 40, 41 but have not been assessed in a study of long‐term cessation in pregnancy. Thus, this study examined potential cigarette dependence related predictors of smoking cessation at 4 weeks post‐quit and end‐of‐pregnancy in a rigorously conducted large trial of a smoking cessation intervention during pregnancy among women who attempted to quit. It is important to identify dependence variables that predict smoking abstinence during pregnancy so that we can target interventions most effectively at women who most need them and understand more clearly the response to interventions among women with varying levels of dependence.

The present study aimed to contribute to the evidence for predictors of smoking cessation during pregnancy by employing a large clinical sample that made a definite quit attempt. This sample enabled a robust test of the predictive ability of baseline measures of cigarette dependence when controlling for a range of socio‐demographic variables through applying a strict criterion for abstinence, involving continuous smoking from the quit date onwards, supported by biochemical verification 4 weeks after the target quit date and at the end of pregnancy.

## Materials and methods

### Participants

**Figure 1:**
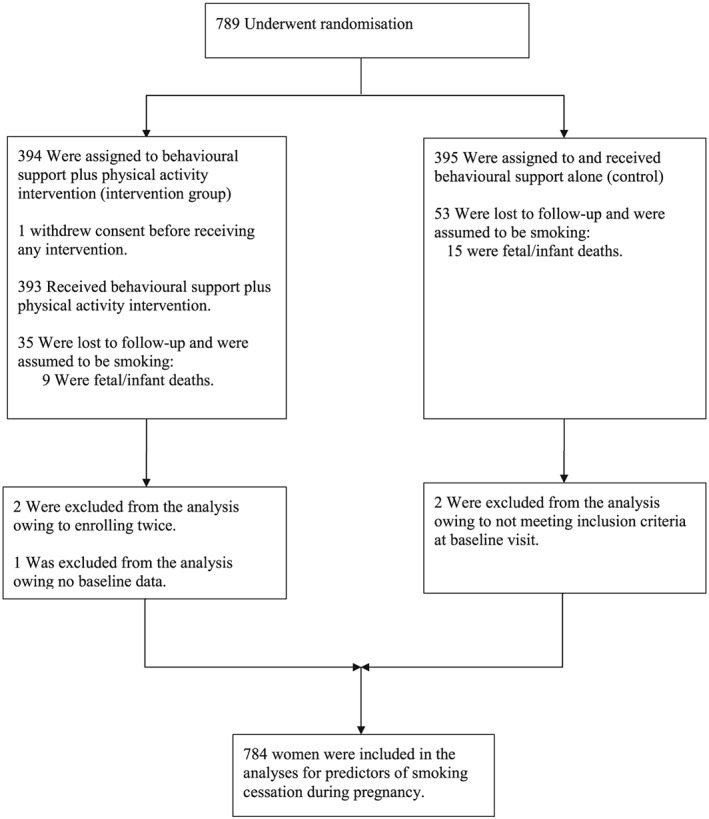
Numbers of participants who were enrolled into the study and included in the analysis

This study is based on the secondary analysis of data from a randomized controlled trial of a physical activity intervention for smoking cessation in pregnancy 42. Of the 8096 recorded as smokers at the first antenatal clinic visit in 13 National Health Service hospitals in southern England, a sample of 789 women who could be contacted fulfilled the inclusion criteria and were willing to participate, were randomized, using random permuted blocks of random size stratified by recruitment centre in a 1 : 1 ratio, to either the physical activity group (*n* = 394) or control. Five women were excluded, two women were enrolled twice in their second pregnancies (we removed their second enrolment), two women were ineligible at their baseline visit but had been randomized erroneously and one woman withdrew consent before providing baseline data. Seven hundred and eighty‐four eligible participants aged 16–50 years, with 10–24 weeks gestation, currently smoking at least one cigarette daily, and prepared to quit smoking 1 week after enrolment, were included in this analysis (Fig. 1).

### Trial protocol

The full protocol for the trial, approved by the Wandsworth NHS Research Ethics Committee, is published elsewhere 43. All participants provided written informed consent. At enrolment, participants were randomized to six sessions of behavioural support alone (control) or this support plus a physical activity (PA) intervention, combining 14 sessions of supervised treadmill exercise and PA consultations. The women were advised to be active for at least 10 minutes at a time, progressing towards 30 minutes of activity on at least 5 days a week. All participants made a quit attempt; they began preparation for quitting at their first treatment session, attempted to quit approximately 1 week after this first session and attended a treatment session on their quit day.

### Baseline measures

The following demographic, psychological and smoking characteristics available at baseline were considered for assessment as potential predictors of smoking cessation: age, ethnicity, body mass index (BMI), marital status, parity, gestational age, gestational interval between baseline and end of pregnancy, study centre, randomization groups (physical activity versus control), alcohol consumption 44, self‐reports of moderate–vigorous‐intensity physical activity (MVPA) in the previous week 45, age at full‐time education, occupation, Edinburgh postnatal depression scale (EPDS) 46 score, partner smoking status, number of cigarettes smoked per day before pregnancy, number of cigarettes smoked per day at baseline, smoking status in previous pregnancy, FTCD score 30 (plus the scores for the HSI and non‐HSI components of the FTCD), expired CO level [parts per million (p.p.m.)] 35 and weekly smoking urges 36. The FTCD (scored 0–10) consists of six items: number of cigarettes smoked per day 10 or less = 0, 11–20 = 1, 21–30 = 2, 31 or more = 3; time to first cigarette of the day (60+ minutes = 0, 31–60 minutes = 1, 6–30 minutes = 2, 0–5 minutes = 3); difficulty not smoking in no‐smoking areas (no = 0, yes = 1); which cigarette would the smoker most hate to give up scored (‘first of the morning’ = 1, others = 0); smoke more frequently in first hours after waking (no = 0, yes = 1); and smoke when ill in bed (no = 0, yes = 1). Higher FTCD scores indicate greater cigarette dependence. The first two FTCD items make up the HSI, scored 0–6 38. Weekly smoking urges (scored 0–10) consists of the combined ratings of strength and frequency of urges 36, 37. The ratings of strength are: no urges = 0, slight = 1, moderate = 2, strong = 3, very strong = 4 and extremely strong = 5; and frequency: not at all = 0, a little of the time = 1, some of the time = 2, a lot of the time = 3, almost all the time = 4 and all the time = 5. As well as the ‘combined’ measure, we examined the frequency and strength of urges measures separately as predictors of abstinence.

### Smoking cessation measures

The outcomes were self‐reported continuous smoking abstinence from quit date to 4 weeks post‐quit and from quit date to end‐of‐pregnancy. Following guidelines, 1West and colleagues' 36 guideline for assessing smoking abstinence advises using self‐report of smoking abstinence over the whole follow‐up period allowing up to five cigarettes in total, with biochemical verification of abstinence, at least, at the end of the follow‐up period. temporary, brief smoking lapses of up to five cigarettes (on up to five occasions) were permitted 47. Biochemical validation of self‐reports was undertaken at 4 weeks post‐quit and end‐of‐pregnancy and concentration of either exhaled CO (< 8 p.p.m.) or salivary cotinine (< 10 ng per millilitre) was used to validate abstinence; if both measures were available, both were required.

### Statistical analysis

Baseline characteristics of the sample were summarized using descriptive statistics. The main aim of the analysis was to understand the association between measures of cigarette dependence and smoking cessation outcomes. In all the following random‐effect logistic regression analyses the dependent variables were smoking cessation at 4 weeks after the quit day and at end‐of‐pregnancy. First, we conducted analysis adjusted for the random effect of study centre to explore the associations between cigarette dependence baseline variables (i.e. scores for the FTCD and the HSI and non‐HSI components of the HSI, number of cigarettes smoked per day, expired CO level and ratings for urges to smoke) and the smoking cessation outcomes. The standardized *Z*‐scores of these variables were used to facilitate the mutual comparison of their effect sizes. We then identified baseline socio‐demographic/health behaviour factors that were associated significantly with smoking cessation by using random‐effect logistic regression analyses. We conducted likelihood ratio tests to assess the statistical significance. For the continuous variables in the random‐effect logistic regression model, it was assumed that the log odds of smoking cessation were related linearly to the continuous predictor. To assess this assumption and determine whether each variable would be best added to the model as a continuous or as a categorical variable, we used the likelihood ratio test (e.g. age at leaving full‐time education was divided into quintiles and the model fits were compared when it was fitted as a categorical variable or as a linear trend).

Next, we used a series of random‐effect logistic regression models to examine the independent associations between each measure of dependence and the cessation outcomes, when adjusting for potential socio‐demographic/health behaviour factors that were shown to be associated significantly (*P* < 0.05) with smoking cessation in the univariate analysis and gestational interval between baseline and end of pregnancy, while allowing for the variability across the study centre and treatment effect. We did not fit a model containing multiple measures of dependence because the measures would be expected to be correlated with each other, leading to potential multicollinearity, and the intention of the analysis was to assess whether all these measures predict smoking cessation outcomes, rather than assessing the independence of their effects. We used adjusted odds ratios [(OR), 95% confidence interval (CI)] and area under the receiver operating characteristic (ROC) curve as a post‐estimation measure of model fit to determine which of the predictors provide higher adjusted effect size and predictive validity. To examine whether the effect of the HSI and non‐HSI is similar to FTCD, we compared their adjusted results from the models.

For 149 (19%) of the participants at 4 weeks post‐quit and 45 (5.7%) at end‐of‐pregnancy, smoking status was not available and it was assumed that they are smoking 47. As a sensitivity analysis, to verify the results obtained in the above analyses, we conducted multiple imputation analyses, which assume instead that data are missing at random. Missing smoking abstinence status was replaced by imputed values using chained equations 48, 49 of logistic regression for smoking cessations at the two follow‐up times. The baseline variables of randomization groups, age at leaving full‐time education, married or living with partner, women with partners who smoke, number of cigarettes smoked daily before pregnancy, FTCD score, any current alcohol use and self‐reporting MVPA > 150 minutes per week and study centre were used as explanatory variables in the imputation models. Three missing values in CO were also replaced by imputed values using linear regression models in the chained equations. We created 20 imputed data sets and conducted the same analyses as above to explore the predictors of smoking cessation in the imputed data sets. The imputation‐specific results of the predictors were combined using Rubin's rules 50. All statistical analyses were conducted using Stata version 12.

## Results

### Baseline characteristics

A summary of the baseline characteristics and smoking abstinence of the sample is provided in Table 1. Seven hundred and eighty‐four participants were included in the analysis; 111 (14%) and 55 (7%) achieved continuous abstinence at 4 weeks and at the end of pregnancy, respectively. The participants were, on average, aged 28 years and 16 weeks pregnant, the majority were married or living with partner, Caucasian and primiparity. Before pregnancy all participants were reasonably heavy smokers, smoking a median of 20 cigarettes per day, and almost half had smoked in a previous pregnancy. At baseline, they still smoked a median of 10 cigarettes per day. At baseline, a quarter reported drinking any alcohol, more than two‐thirds reported ≥ 150 minutes weekly of moderate–vigorous intensity physical activity. For the dependence measures there was evidence of multi‐collinearity, such that the correlation coefficients between FTCD score and number of cigarettes smoked per day, expired CO level and ratings for urges to smoke were 0.411, 0.556 and 0.386, respectively (all at *P* < 0.001).

**Table 1:** Baseline characteristics of the sample.

*Variables*	(n = 784) Mean (SD)
Age (years)	27.5 (6.3)
Age at leaving full‐time education (years)^a^	17.7 (2.9)
Weight (kg)	70.0 (15.0)
Body Mass Index 9 (kg/m)^2^ b	26.1 (5.3)
Gestational age (weeks)	15.6 (3.3)
Gestational interval between baseline and end‐of‐pregnancy (weeks)	23.3 (4.0)
	*Median (IQR)*
Number of cigarettes smoked daily before pregnancy	20 (12–20)
Number of cigarettes smoked per day at baseline	10 (5–13
Fagerström Test of Cigarette Dependence score	4 (2–5)
Heaviness of Smoking Index score^c^	2 (1–3)
Non‐Heaviness of Smoking Index score^d^	2 (1–3)
Expired carbon monoxide (CO) level (p.p.m.)^e^	10 (6–14)
Urge to smoke score^f^	6 (4–8)
Frequency of urge to smoke	3 (2–4)
Strength of urge to smoke	3 (2–4)
Self‐reported of weekly MVPA (minutes)	210.0 (130–355)
	*No. (%)*
Randomization group (physical activity)	391 (49.9)
Married or living with partner	451 (58)
Women with partners who smoke	511 (65.2)
Caucasian^g^	607 (77)
Professional/managerial occupation	99 (13)
Smoked in a previous pregnancy	379 (48)
Edinburgh Postnatal Depression Scale score > 15	83 (10.6)
Self‐reporting MVPA ≥ 150 minutes/week	548 (69.9)
Primiparity^h^	420 (53.6)
Previous preterm birth^i^	129 (16.5)
Any current alcohol use	201 (25.6)

MVPA = moderate–vigorous intensity physical activity; IQR = interquartile range; p.p.m. = parts per million.

a For 41 women, current age was considered as age at full‐time education, as they were still in full‐time education.

b For three women, weight/body mass index (BMI) at their first antenatal booking visit was used as baseline weight/BMI, as it was not recorded for them at baseline.

c Composed of two Fagerström Test of Cigarette Dependence items (i.e. time to first cigarette of the day and number of cigarettes usually smoked per day).

d Comprising four Fagerström Test of Cigarette Dependence items other than the two items of Heaviness of Smoking Index (HIS).

e Carbon monoxide (CO) was not recorded for three participants.

f Urge to smoke score = frequency of urges + strength of urges.

g Race or ethnic group was self‐reported and categorized according to standard UK census categories.

h Primiparity was defined as the first‐time pregnancy progressing beyond 24 weeks.

i Previous preterm birth was defined as any previous pregnancy that lasted from 24 to 37 weeks.

### Random‐effect logistic analysis to explore the predictors

In the univariate regression analyses (see Table 3), the significant cigarette dependence‐related predictors of smoking abstinence at both 4 weeks and end‐of‐pregnancy were: lower score for the FTCD and its two components (i.e. HSI and non‐HSI), lower number of cigarettes smoked daily, lower expired CO level and lower score for urges to smoke (for both ‘combined’ measure and for separate measures for frequency and strength of urges). When we assessed whether all the continuous independent variables were fitted appropriately as linear effects in the logistic regression analyses, the likelihood ratio test suggested no evidence of departure from linear effects.

**Table 2:** Random‐effect logistic regression analyses^a^ for socio‐demographic/health behaviour predictors of smoking cessation at 4 weeks post‐quit and the end‐of‐pregnancy (*n* = 784)^b^.

*Measures*	4 Weeks post‐quit		End of pregnancy	
	OR (95% CI)	P‐values	OR (95% CI)	P‐values
Age (years)^c^	1.02 (0.99, 1.06)	0.172	1.02 (0.98, 1.06)	0.377
Age at leaving full‐time education (years)^c^, ^d^	1.07 (1.02, 1.14)	**0.021**	1.10 (1.01, 1.17)	**0.022**
Body Mass Index (kg/m^2^)^c^, ^e^	1.00 (0.97, 1.04)	0.779	1.02 (0.97, 1.07)	0.475
Married or living with partner	2.01 (1.29, 3.12)	**0.002**	1.91 (1.04, 3.49)	**0.036**
Primiparity^f^	1.13 (0.75, 1.70)	0.549	1.20 (0.69, 2.10)	0.520
Women with partners who smoke	0.86 (0.54, 1.37)	0.529	0.96 (0.51, 1.83)	0.909
Edinburgh Postnatal Depression Scale score > 15	1.20 (0.63, 2.28)	0.573	0.84 (0.32, 2.17)	0.713
Self‐reporting MVPA ≥ 150 minutes/week	2.64 (1.53, 4.57)	**<0.001**	1.77 (0.89, 3.50)	0.102
Alcohol use	1.17 (0.74, 1.83)	0.506	1.68 (0.94 2.99)	0.080
Gestational age (weeks)	1.00 (0.94, 1.06)	0.917	0.95 (0.87, 1.04)	0.248
Gestational interval between baseline and end of pregnancy	1.00 (0.95, 1.05)	0.923	1.08 (1.00, 1.16)	0.051
Living in a deprived area	0.75 (0.50, 1.14)	0.180	1.08 (0.61, 1.91)	0.788
Caucasian^g^	0.87 (0.54, 1.40)	0.565	1.05 (0.54, 2.050)	0.879
Occupation (managerial versus all others)	1.09 (0.60, 1.99)	0.765	1.37 (0.65, 2.91)	0.406

*P*‐values shown in bold text represent significant associations at *P*<0.05. MVPA = moderate–vigorous intensity physical activity; OR = odds ratio; CI = confidence interval.

a Adjusted for the random effect of study centre.

b For 149 and 44 participants at 4 weeks post‐quit and end of pregnancy, respectively, the outcome was missing and it was assumed that they are smoking.

c The ORs reflect an effect of per unit change of the independent variable on smoking cessation outcome.

d For 41 women, current age was considered as age at full‐time education as they were still in full‐time education.

e For three women, weight/body mass index at their first antenatal booking visit was used as baseline weight/body mass index as it was not recorded for them at baseline.

f Primiparity was defined as the first‐time time pregnancy progressing beyond 24 weeks.

g Race or ethnic group was self‐reported and categorized according to standard UK census categories.

**Table 3:** Random‐effect logistic regression analyses to assess the ability of each cigarette dependence related measure in predicting smoking cessation (*n* = 784).^a^

	Random‐effect logistic regression analyses^b^		Random‐effect multiple logistic regression models^c^			
Measures	4 Weeks post‐quit	End of pregnancy	4 Weeks post‐quit		End of pregnancy	
	OR (95% CI)^d^	OR (95% CI)^d^	Adjusted OR (95% CIs)^d^	AUROC (95% CIs)	Adjusted OR (95% CIs)^d^	AUROC (95%CIs)
Fagerström Test of Cigarette Dependence score	0.56 (0.45, 0.70)	0.58 (0.43, 0.77)	0.59 (0.47, 0.74)	0.702 (0.648–0.756)	0.60 (0.45, 0.81)	0.673 (0.617, 0.730)
Heaviness of Smoking Index score^e^	0.59 (0.48, 0.73)	0.60 (0.45, 0.80)	0.63 (0.51, 0.79)	0.692 (0.636, 0.747)	0.65 (0.48, 0.87)	0.666 (0.609, 0.723)
Non‐Heaviness of Smoking Index score^f^	0.63 (0.51, 0.78)	0.64 (0.48, 0.86)	0.64 (0.51, 0.79)	0.694 (0.641, 0.747)	0.65 (0.48, 0.88)	0.660 (0.504, 0.717)
Expired carbon monoxide (CO) level (p.p.m.)^g^	0.55 (0.42, 0.72)	0.55 (0.38, 0.79)	0.54 (0.41, 0.71)	0.711 (0.659, 0.763)	0.55 (0.37, 0.80)	0.681 (0.628, 0.735
Number of cigarettes smoked per day at baseline	0.62 (0.48, 0.80)	0.66 (0.47, 0.92)	0.65 (0.51, 0.84)	0.690 (0.633, 0.746)	0.70 (0.49, 0.98)	0.650 (0.593, 0.709)
Urge to smoke score^h^	0.78 (0.63, 0.96)	0.66 (0.50, 0.88)	0.82 (0.66, 1.01)	0.672 (0.617, 0.727)	0.69 (0.51, 0.93)	0.630 (0.572, 0.687)
Frequency of urge to smoke	0.74 (0.60, 0.91)	0.66 (0.49, 0.88)	0.79 (0.63, 0.98)	0.675 (0.620, 0.730)	0.69 (0.51, 0.94)	0.637 (0.580, 0.693)
Strength of urge to smoke	0.87 (0.71, 1.07)	0.74 (0.56, 0.98)	0.90 (0.72, 1.11)	0.664 (0.610, 0.719)	0.77 (0.57, 1.03)	0.623 (0.566, 0.679

a For 149 and 44 participants at 4 weeks post‐quit and end of pregnancy, respectively, the outcome was missing and it was assumed that they are smoking.

b Adjusted for the random effect of study centre.

c Adjusted for the potential confounders of age at leaving full‐time education, married or living with partner, self‐reporting MVPA ≥ 150 minutes/week, randomization groups, gestational interval between baseline and end of pregnancy, and random effect of study centre in the random‐effect logistic regression model. The mixed‐ effect multiple logistic models are separate models for each dependence measure and do not include the other dependence measures.

d Odds ratio (OR) [95% confidence intervals (CIs)] = the ORs (95% confidence intervals) reflect lower odds of abstinence for per unit standard deviation increase in values of the predictors.

e Composed of the two Fagerström Test of Cigarette Dependence items (i.e. time to first cigarette of the day and number of cigarettes usually smoked per day).

f Comprised of the four Fagerström Test of Cigarette Dependence items other than the two items of Heaviness of Smoking Index.

g CO was not recorded for three participants.

h Weekly urges to smoke score = composite of frequency of urges plus strength of urges; p.p.m. = parts per million.

AUROC = Area Under the Receiver Operating Characteristic (ROC) curve.

Of the socio‐demographic/health behaviour variables, higher age at leaving full‐time education and married or living with partner were associated significantly with smoking abstinence at both 4 weeks and end‐of‐pregnancy; self‐reporting MVPA ≥ 150 minutes per week reached the 10% level of significance for end‐of‐pregnancy abstinence and was significant at the 5% level for 4 weeks, gestational interval between baseline and end‐of‐pregnancy approached significance at end‐of‐pregnancy (see Table 2). Therefore we adjusted the effect of each cigarette dependence‐related predictor for these four variables while also allowing for variability across the study centres and treatment effect in the models (see Table 3).

### Multiple random‐effect logistic regression analyses for each of the dependence measures

In multiple logistic regression analyses, each measure of cigarette dependence remained as a significant predictor of smoking abstinence at 4 weeks. Adjusted OR (95% CI) for a unit standard deviation increase in FTCD score was 0.59 (0.47–0.74), HSI 0.64 (0.51–0.79), non‐HSI 0.64 (0.51–0.79), expired CO 0.54 (0.41–0.71), number of cigarettes smoked per day 0.65 (0.51–0.84) and frequency of urges to smoke 0.79 (0.63–0.98); and at end‐of‐pregnancy for FTCD 0.60 (0.45–0.81), HSI 0.65 (0.48–0.87), non‐HSI 0.65 (0.48–0.88), expired CO 0.55 (0.37–0.80), number of cigarettes smoked per day 0.70 (0.49–0.98) and frequency of urges to smoke 0.69 (0.51–0.93). The combined score of frequency and strength of urges to smoke was also a significant predictor of abstinence at end‐of‐pregnancy and approached significance at 4 weeks (see Table 3) while strength of urges to smoke did not predict abstinence significantly at either time‐point. In all cases, higher levels of the measures of cigarette dependence were associated with worse outcomes for abstinence. The values of area under the ROC curve for the model's performance showed that the predictive validity for all the dependence measures was very similar (see Table 3).

### Sensitivity analyses using multiple imputations

When we used multiple imputations as an alternative way of dealing with missing outcomes data, the results for all analyses were very similar. In particular, at end‐of‐pregnancy the adjusted pooled OR (95% CI) for each association of the scores for FTCD and its two subscales (i.e. HSI and non‐HSI), expired CO level, number of cigarettes smoked daily and the score for urges to smoke were: 0.58 (0.43–0.78), 0.61 (0.45–0.83), 0.64 (0.48–0.86), 0.53 (0.36–0.78), 0.68 (0.48–0.96) and 0.74 (0.55–0.98), respectively.

## Discussion

Cigarette dependence, measured by the FTCD or by its HSI or non‐HSI components, expired CO level, cigarette consumption or frequency of urges to smoke predicted smoking cessation significantly at 4 weeks post‐quit during pregnancy and at end‐of‐pregnancy.

The finding for FTCD predicting abstinence is consistent for observations with non‐pregnant smokers 23, 27, 31. In our study, the predictive ability of the two components of FTCD (i.e. HSI and non‐HSI) and their effect sizes were similar to FTCD; therefore, for economy, it might be better to use the HSI, composed of only two items, for assessing cigarette dependency in pregnant smokers. The finding that lower expired CO levels predicted cessation is consistent with the previous finding for saliva cotinine 28, another biochemical marker of abstinence, and for CO levels predicting abstinence at 6 months postpartum 51, as well as with findings for non‐pregnant smokers 34. The finding for number of cigarettes smoked per day is consistent with the results of lower number of cigarettes smoked per day before pregnancy predicting cessation 18. Thus, number of cigarettes smoked per day or expired CO may also be considered as valid brief predictors of smoking cessation during pregnancy. The result for number of cigarettes smoked per day is important in pregnancy, as almost all women smokers who do not quit reduce their smoking rate significantly when they find that they are pregnant (by about 50% in this study). Thus, despite serious cutting down, the smoking rate still predicted abstinence. Urges to smoke have not been tested previously as a predictor of smoking cessation during pregnancy, and in this study frequency of urges to smoke showed significant results at the two times and the combined score for urges was also significant at end‐of‐pregnancy, which is consistent with the results for studies in non‐pregnant populations 37, 40, 41.

Our review of the literature found that data for the majority of studies reporting predictors of smoking cessation in pregnancy were from observational studies, and only a few used data from clinical trials. Of the studies which used biochemically validated trial data, only two had a large sample size. Power analysis was not conducted for this study as it was based on secondary analysis, but our study had a large sample size with biochemically validated continuous smoking cessation from quit day through to end‐of‐pregnancy, with all participants making a quit attempt. Thus, our study was conducted rigorously, using a strict criterion for abstinence, and the use of a strict abstinence criterion is important when testing associations with factors promoting or undermining success of a quit attempt. Weaker outcome measures, such as point prevalence abstinence, are less useful, because someone can have a full relapse to smoking on one or more occasions and still be counted as abstinent, thus blurring the distinction between predicting quit attempts and quit success. The quit rate of 7% in this study was lower than in many previous pregnancy trials, examining predictors with less rigorous abstinence criteria, but was similar to a study using comparable abstinence criteria 28. Our study had a large sample size with greater power for the analyses compared with most previous studies. We used careful multivariable analysis methods, with an efficient sensitivity analysis for missing smoking status using multiple imputations, which investigated adjusted associations of the measure of cigarettes dependence with abstinence. Compared with most previous studies, we included smokers with a wider range of levels of cigarette dependence (with eligible women only needing to be smoking at least one cigarette a day at baseline); therefore, with regard to dependence, the findings are likely to be applicable to pregnant smokers in general.

This study is potentially limited in terms of the representativeness of the sample. First, the participants were recruited predominantly in London, where the smoking rates tend to be lower than the rest of the country. Secondly, some of the women recorded as smokers at the antenatal clinics could not be contacted; of those who were contacted, some declined the offer of joining the study and some were excluded due to the exclusion criteria of the trial, although there were few exclusion criteria 43. We were unable to compare the characteristics of those who were recruited with those who were not. We recruited women who mainly reported being physically active at baseline and who, therefore, might be more motivated to quit than less active women; this is likely to be because active women were attracted to a trial promoting physical activity in a health‐care setting. Ten per cent of women recorded as smokers at the first antenatal booking visit were recruited, which was the target recruitment rate and is similar to rates for other large UK trials of smoking cessation in pregnancy 52, 53. Quit rates were lower than for pregnancy trials with less rigorous outcome measures, but were similar to those for studies using comparable outcomes 4. The women were generally representative of women who smoke 54, and the findings are likely to be generalizable to primary and secondary care settings.

We have reported elsewhere 42 that there was no significant effect of the multi‐session treatment on smoking cessation; however, we adjusted the results for the treatment effect in this study. The intervention group had to change two behaviours (i.e. smoking and physical activity) simultaneously, while also coping with being pregnant and attending multiple treatment sessions; this could have a negative impact on cessation, which may then have an effect on the predictive ability of the dependency measures. Although this study considered a broad range of variables that might have an impact upon smoking cessation there are other variables which we did not include, which have been found to predict cessation in pregnancy and which might be important, such as whether the pregnancy is planned, exposure to environmental tobacco smoke, exposure to patient education methods, perceived social support, stressful life events in early pregnancy, use of illicit substances before pregnancy, motivation to quit and nausea during pregnancy 19, 20, 21, 22.

These findings are important for public health policy, as they highlight the importance of cigarette dependence for smoking cessation in pregnancy, demonstrate that multiple facets of dependence are likely to play a role in cessation and identify dependence measures that are clinically quick to administer to tailor cessation treatments. As cigarette dependence appears to be a predictor of smoking cessation during pregnancy, interventions need to focus upon supporting quit attempts among those who seek treatment and are more highly dependent (e.g. through helping women avoid and manage urges to smoke). Assessment of dependence during pregnancy is crucial, so that appropriate support is provided to those women who are most dependent, with increased intensity of support, including higher doses and longer durations of nicotine replacement, for those with higher dependence 52. The finding that the non‐HSI part of the FTCD was predictive of abstinence suggests that, besides the commonly used HSI, the non‐HSI may also be important.

## Conclusion

These findings show that, in a trial of a smoking cessation intervention, higher levels of several common baseline measures of cigarette dependence, including number of cigarettes smoked per day, scores for FTCD and frequency of urges and level of expired CO, all predicted smoking abstinence in the short term during pregnancy and at end‐of‐pregnancy with very similar predictive validity. In research studies or in clinical settings, it may be most practicable to include either of the brief components of FTCD (i.e. HSI and non‐HSI) rather than the full FTCD. Studies are needed to investigate these and other measures of cigarette dependence as predictors of smoking abstinence in further trials and in population‐based studies.

## Declaration of interests

In the past 3 years, P.A. has conducted one day of consultancy for Pfizer concerning general smoking cessation advice; R.W. has undertaken research and consultancy for Pfizer and GlaxoSmithKline, that develop and manufacture smoking cessation; T.C. has been paid for speaking at one educational event that was part‐sponsored by Pierre Fabre Laboratories, France, that manufactures nicotine replacement therapy; R.W. is an unpaid trustee of the stop smoking charity QUIT and an unpaid director of the National Centre for Smoking Cessation and Training. This project was funded by the National Institute for Health Research Health Technology Assessment Programme (project number 07.01.14). The open access publication has been funded by Cancer Research UK.
